# Machine learning algorithms for the early detection of bloodstream infection in children with osteoarticular infections

**DOI:** 10.3389/fped.2024.1398713

**Published:** 2024-12-11

**Authors:** Yuwen Liu, Yuhan Wu, Tao Zhang, Jie Chen, Wei Hu, Guixin Sun, Pengfei Zheng

**Affiliations:** ^1^ Department of Orthopaedic Surgery, Children’s Hospital of Nanjing Medical University, Nanjing, China; ^2^ State Key Laboratory for Novel Software Technology, Nanjing University, Nanjing, China; ^3^ Department of Orthopaedic Surgery, Qinghai Province Women and Children’s Hospital, Xining, China; ^4^ Department of Orthopaedic Surgery, Wuxi Children’s Hospital, Wuxi, China; ^5^ Department of Traumatic Surgery, Shanghai East Hospital, Nanjing Medical University, Nanjing, China

**Keywords:** bloodstream infection, osteoarticular infection, machine learning, children, diagnosis

## Abstract

**Background:**

Bloodstream infection (BSI) poses a significant life-threatening risk in pediatric patients with osteoarticular infections. Timely identification of BSI is crucial for effective management and improved patient outcomes. This study aimed to develop a machine learning (ML) model for the early identification of BSI in children with osteoarticular infections.

**Materials and methods:**

A retrospective analysis was conducted on pediatric patients diagnosed with osteoarticular infections admitted to three hospitals in China between January 2012 and January 2023. All patients underwent blood and puncture fluid bacterial cultures. Sixteen early available variables were selected, and eight different ML algorithms were applied to construct the model by training on these data. The accuracy and the area under the receiver operating characteristic (ROC) curve (AUC) were used to evaluate the performance of these models. The Shapley Additive Explanation (SHAP) values were utilized to explain the predictive value of each variable on the output of the model.

**Results:**

The study comprised 181 patients in the BSI group and 420 in the non-BSI group. Random Forest exhibited the best performance, with an AUC of 0.947 ± 0.016. The model demonstrated an accuracy of 0.895 ± 0.023, a sensitivity of 0.847 ± 0.071, a specificity of 0.917 ± 0.007, a precision of 0.813 ± 0.023, and an F1 score of 0.828 ± 0.040. The four most significant variables in both the feature importance matrix plot of the Random Forest model and the SHAP summary plot were procalcitonin (PCT), neutrophil count (N), leukocyte count (WBC), and fever days.

**Conclusions:**

The Random Forest model proved to be effective in early and timely identification of BSI in children with osteoarticular infections. Its application could aid in clinical decision-making and potentially mitigate the risk associated with delayed or inaccurate blood culture results.

## Introduction

Blood culture serves as a crucial diagnostic tool for infectious diseases in pediatric patients. Positive results indicate bacteremia or BSI, prompting appropriate antibiotic treatment. BSI, at times, coincide with osteoarticular infections (1–3), potentially leading to multifocal haematogenous osteomyelitis or sepsis, with septic shock being a significant cause of mortality (4, 5). However, blood culture results are susceptible to false positives resulting from contamination and false negatives due to prior antibiotic usage (6–8). Moreover, the process typically takes 12 to 48 h to yield results, exposing pediatric patients to potential risks (9–11).

To enhance the reliability and speed of BSI detection, several approaches have been developed. Serum marker tests such as C-reactive protein (CRP) and PCT offer quick and straightforward methods aiding in early diagnosis and treatment monitoring. However, they lack specificity and can be influenced by various factors (12). Polymerase chain reaction (PCR) boasts high sensitivity and specificity, facilitating the rapid identification of pathogenic microbial DNA or RNA and providing faster results. Nevertheless, PCR is relatively expensive and can be affected by sample quality and detection methodology (13). Pathogen gene microarray testing, despite its capability to simultaneously detect multiple pathogens, remains less accessible due to its high cost and the requirement for specialized equipment and expertise (14).

ML stands as the cornerstone technology of artificial intelligence (AI) today, facilitating intricate decision-making processes through learning. Its application in healthcare is increasingly prevalent, enabling analysis of variables pertinent to clinical outcomes and supporting various clinical activities. Compared to conventional regression analysis, ML offers superior modeling of complex relationships (15, 16). Figueroa-Phillips et al. (17) developed a clinical prediction model for central line-associated BSI, aiming to mitigate unnecessary hospital admissions and antibiotic usage. Similarly, Tabaie et al. (18) utilized deep learning models to integrate structured and unstructured data, achieving timely prediction of BSI among children with central venous lines. While these ML models demonstrated convenience and rapidity in BSI detection, they were constrained by variations in variable selection depending on the disease within the study population, rendering them less suitable for pediatric patients with osteoarticular infections. Thus, there arises a necessity to develop a diagnostic model for BSI tailored specifically to ensure the medical safety of pediatric patients with osteoarticular infections.

The objective of this study was to harness ML algorithms to craft an AI model customized for the early detection of BSI linked with osteoarticular infections in the pediatric population.

## Materials and methods

### Patient information

In this study, pediatric patients diagnosed with osteoarticular infections were admitted to three hospitals in China between January 2012 and January 2023. The inclusion criteria were as follows: ① Diagnosis of osteoarticular infections, including osteomyelitis and septic arthritis; ② Availability of complete original data, encompassing medical history, laboratory test results, and imaging data. Exclusion criteria included: ① Neonatal patients (≤28 days old); ② Inconsistent results of blood and puncture fluid cultures; ③ Chronic osteoarticular infections, including the formation of dead bone or sinus tracts; ④ Patients with tuberculosis, syphilis and fungal infections were excluded; ⑤ Suspected cases of aseptic arthritis or tumor-related lesions; ⑥ Patients with uncertain health status or immune deficiency.

**Figure 1:**
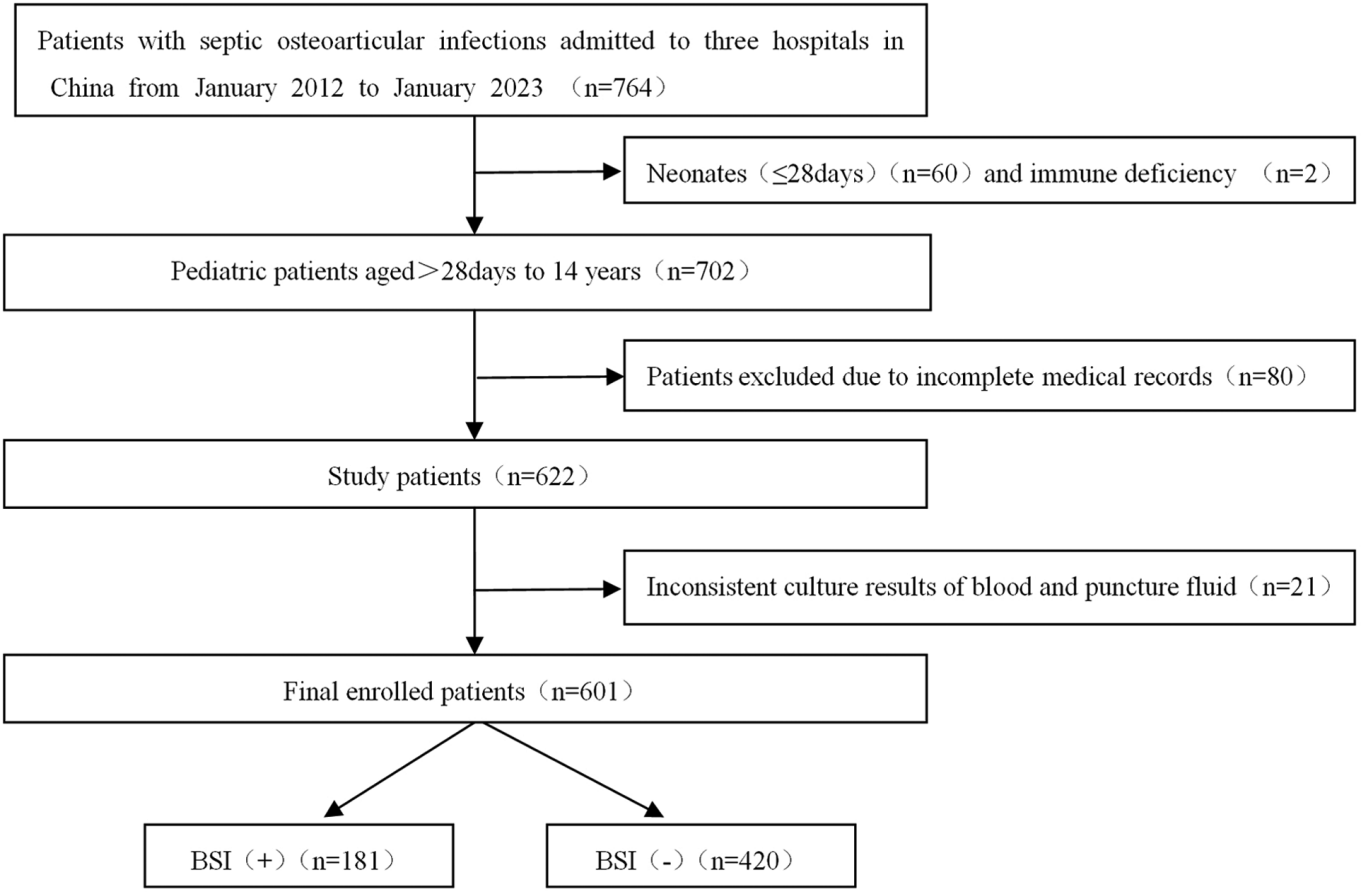
Flow chart of study inclusion.

Data from 764 patients were initially collected using the electronic medical record system. Subsequently, 601 patients with complete data preservation were ultimately enrolled in the study. The age range of the patients spanned from over 28 days to 14 years old, mean 6.08 ± 3.92 years. The detailed screening process was depicted in Figure 1.

### Bacterial culture

The presence and type of BSI were determined based on a positive blood culture result. Bacterial cultures of both the blood and puncture fluids were systematically conducted for all cases.

Blood culture: 1–3 ml of peripheral venous blood was collected and injected into a paediatric aerobic blood culture bottle, which was placed in the BACT/ALERT 3D blood culture instrument. If there was bacterial growth, the instrument would alarm and microbiology staff would swab, stain, microscope the bottle and transfer the bacteria to a Columbia Blood Plate and a Chocolate Plate and incubate the plate for 24 h at 5% carbon dioxide and 35°C. After colony growth, the bacteria were identified and drug susceptibility testing was performed using a VITEK 2 COMPACT instrument.

Puncture fluid culture: puncture fluid was inoculated directly into Columbia Blood and China Blue plates and incubated for 24 h at 35°C in 5% carbon dioxide. After colony growthed, bacterial identification and drug susceptibility testing were performed using a VITEK 2 COMPACT instrument.

To mitigate the effects of sampling contamination, two distinct sets of collection devices and two culture bottles were utilized for specimen collection. Positive bacterial culture was confirmed if the results of both cultures were consistent. In the investigated cohort, blood culture yielded positive results in 202 patients and negative findings in 420 patients. Conversely, puncture fluid culture yielded positive outcomes in 294 patients, while 328 patients yielded negative results. Furthermore, to enhance the consistency of the pathological diagnosis of osteoarticular infections and BSI, 21 patients with disparities in bacterial identification between blood culture and puncture fluid culture results were excluded. The culture results were summarized in Table 1.

**Table 1:** Culture results of blood and puncture fluid.

Pathogens	Blood culture (+) (*n* = 183)	Puncture fluid culture (+) (*n* = 275)
Staphylococcus aureus, *n* (%)	129 (70.5)	211 (76.7)
Methicillin resistant Staphylococcus aureus, *n* (%)	28 (15.3)	37 (13.4)
Pyogenic streptococcus, *n* (%)	5 (2.7)	10 (3.6)
Staphylococcus hominis, *n* (%)	15 (8.2)	9 (3.3)
Haemophilus influenzae, *n* (%)	4 (2.2)	4 (1.5)
Streptococcus pneumoniae, *n* (%)	2 (1.1)	4 (1.5)

### Clinical variables

In this study, sixteen independent variables available for assessment during the initial consultation underwent meticulous scrutiny. The patient's demographic characteristics (age, gender and weight) and clinical history (fever days, peak temperature, recent diseases, symptom duration, symptoms of other systems) were extracted from the medical records. Serum markers, including WBC, N, platelet count (PLT), hemoglobin (Hb), CRP, erythrocyte sedimentation rate (ESR), and PCT, were also extracted. Imaging variable such as bone damage was evaluated using radiography or computed tomography (CT). Symptom duration was defined as the interval between the onset of clinical symptoms and the time of consultation. Recent diseases included trauma or other infectious conditions such as respiratory, gastrointestinal, urinary tract, and soft tissue infections. Symptoms of other systems included cough, diarrhea, vomiting, headache, and convulsion.

### Data preprocessing

Based on the original data, all null data were removed. Data with a z-score above the threshold were considered anomalous and subsequently removed from the dataset. Through this preprocessing procedure, 181 patients in the BSI group and 420 patients in the non-BSI group were included in the study. Peak temperature was classified into five grades based on the degree of fever: 36.3–37.2°C was normal, 37.3–38°C was mild fever, 38.1–39°C was moderate fever, 39.1–41°C was high fever and temperatures exceeding 41°C was super-high fever. Recent diseases were categorised into three conditions: none, trauma, and infection. Symptoms of other systems and bone damage were classified as either yes or no. Ordinal coding was applied to categorical features such as gender, peak temperature, recent diseases, symptoms of other systems, and bone damage.

### Machine learning procedure

The study utilized classification models from Python 3.11.4 and scikit-learn 1.2.2 to construct predictive models by training on the collected data. The models included Logistic Regression, Kneighbors, Gaussian NB, Linear SVC, Decision Tree, SGDC lassifier, Random Forest, and Gradient Boosting Trees. The entire dataset was split into training and testing sets using a 70–30 ratio, with five-fold cross-validation employed during model training to enhance model robustness and mitigate overfitting. Various evaluation metrics, such as accuracy, sensitivity, specificity, precision, and F1 score, were computed for each model. ROC curves were generated, and the AUC was calculated using the trapezoidal method to assess the models' performance. The selection of the optimal model for training was based on both AUC and accuracy. An importance matrix was utilized to illustrate all the features predictive of BSI. The SHAP (19) method was employed to assess the value for each variable within the model based on the training dataset. Additionally, SHAP dependence plots were generated to explain how individual features influence the output of the ML algorithm.

### Statistical analysis

The data analysis was conducted using SPSS 27.0 software (IBM Corp., Armonk, NY, USA). Quantitative data were presented as mean ± standard deviation (SD). Differences in predictive variables between the BSI group and non-BSI group were compared. Continuous variables were compared using the independent *t*-test, while the Chi-square test was utilized for categorical variables. Statistical significance was defined as *P* < 0.05.

## Results

### Participant characteristics

The clinical characteristics of the BSI and non-BSI groups were summarized in Table 2. Variables including age, weight, fever days, peak temperature, recent diseases, WBC, N, CRP, ESR, PCT, and bone damage exhibited statistically significant differences between the two groups (*P* < 0.05). However, variables such as gender, symptom duration, PLT, Hb, and symptoms of other systems did not show statistically significant differences between the two groups (*P* > 0.05).

**Table 2:** Comparison of the clinical characteristics of BSI and non-BSI group.

Variables	BSI (+) (*n* = 181)	BSI (−) (*n* = 420)	*P*-value
Age (years)	6.58 ± 3.53	5.87 ± 3.89	0.04
Gender, *n* (%)			0.6
Male	114 (18.97)	255 (42.43)	
Female	67 (11.15)	165 (27.45)	
Weight (kg)	27.02 ± 14.30	24.56 ± 13.10	0.04
Fever days (days)	5.54 ± 3.57	3.79 ± 2.99	<0.001
Peak temperature, *n* (%)			<0.001
Normal	8 (1.33)	40 (6.66)	
Mild fever	1 (0.16)	39 (6.49)	
Moderate fever	62 (10.32)	228 (37.94)	
High fever	109 (18.14)	113 (18.80)	
Super-high fever	1 (0.16)	0 (0)	
Recent Diseases, *n* (%)			<0.001
None	85 (14.14)	235 (39.10)	
Trauma	64 (10.65)	86 (14.31)	
Infections	32 (5.33)	99 (16.47)	
Symptom duration (days)	6.99 ± 4.57	7.51 ± 5.86	0.24
Serum indicators			
WBC (×10^9^/L)	16.89 ± 4.62	13.07 ± 4.48	<0.001
*N* (×10^9^/L)	12.38 ± 4.33	8.59 ± 4.15	<0.001
PLT (×10^9^/L)	368.52 ± 150.63	384.62 ± 133.02	0.19
Hb (g/L)	113.39 ± 13.97	114.14 ± 13.05	0.53
CRP (mg/L)	90.20 ± 51.88	61.02 ± 51.94	<0.001
ESR (mm/h)	67.23 ± 23.44	57.48 ± 28.27	<0.001
PCT (ng/ml)	1.77 ± 2.38	0.50 ± 1.97	<0.001
Symptoms of other systems			0.07
Yes, *n* (%)	28 (4.66)	92 (15.31)	
no, *n* (%)	153 (25.46)	328 (54.57)	
Bone damage			0.034
yes, *n* (%)	45 (7.49)	141 (23.46)	
no, *n* (%)	136 (22.63)	279 (46.42)	

### Model performance

**Figure 2:**
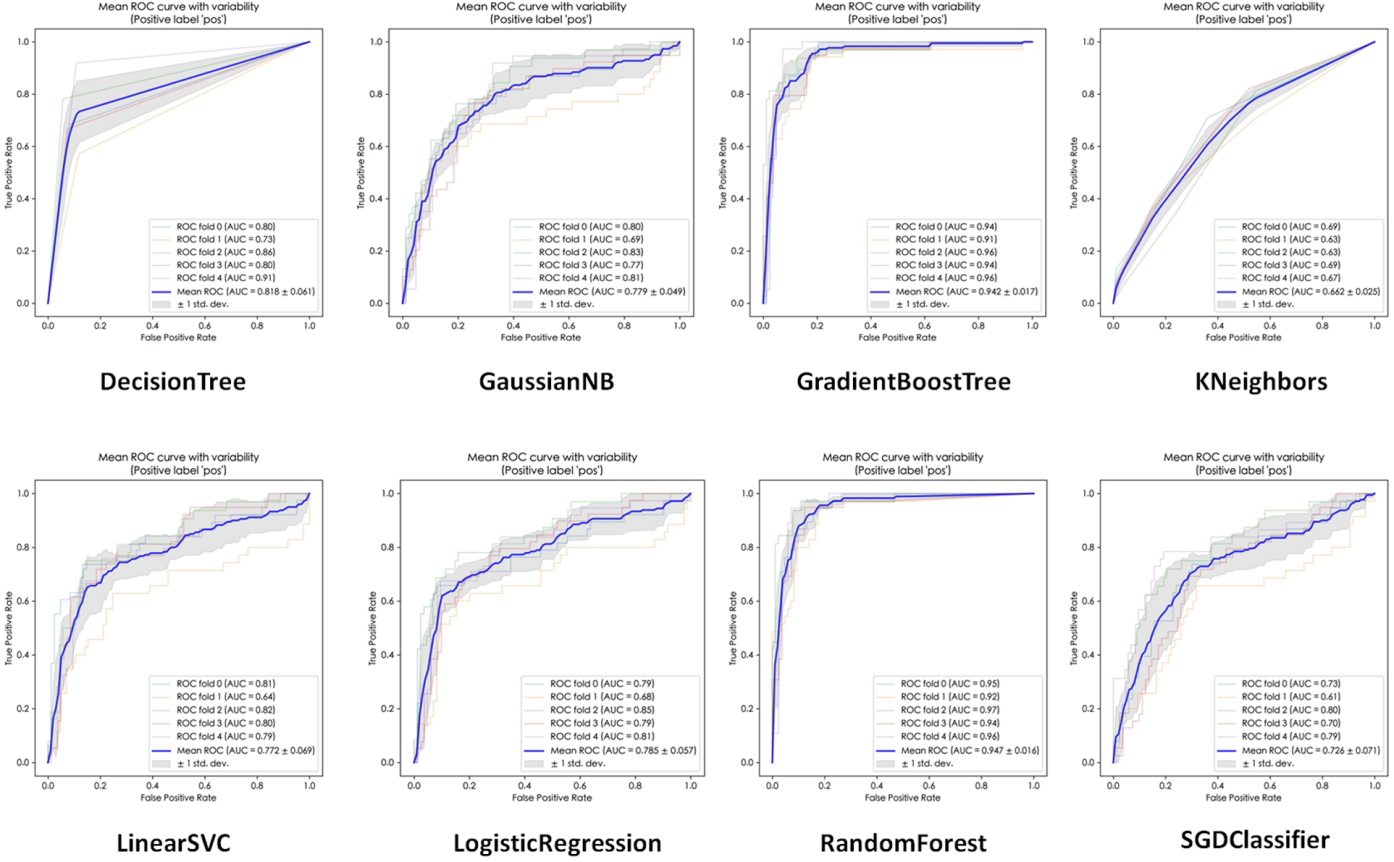
ROC curves of various algorithms.

The performance of the eight ML algorithms were presented in Table 3. Additionally, the ROC curves and micro-average AUC for each model were depicted in Figure 2. Among the models, Random Forest exhibited the highest AUC (0.947 ± 0.016), followed by Gradient Boost Tree (0.942 ± 0.017), Decision Tree (0.818 ± 0.061), Logistic Regression (0.785 ± 0.057), Gaussian NB (0.779 ± 0.049), Linear SVC (0.772 ± 0.069), SGDC lassifier (0.726 ± 0.071), and Kneighbors (0.662 ± 0.025).

**Table 3:** Performance of the various algorithmic models.

Machine learning algorithms	Accuracy	Sensitivity	Specificity	Precision	F1 Score
Logistic Regression	0.802 ± 0.037	0.512 ± 0.085	0.929 ± 0.021	0.754 ± 0.072	0.607 ± 0.072
Kneighbors	0.686 ± 0.020	0.364 ± 0.025	0.824 ± 0.034	0.476 ± 0.067	0.411 ± 0.037
GaussianNB	0.737 ± 0.033	0.282 ± 0.156	0.936 ± 0.053	0.689 ± 0.096	0.370 ± 0.126
LinearSVC	0.697 ± 0.081	0.367 ± 0.347	0.834 ± 0.242	0.480 ± 0.259	0.335 ± 0.240
Decision Tree	0.855 ± 0.041	0.724 ± 0.118	0.912 ± 0.022	0.778 ± 0.058	0.747 ± 0.080
SGD	0.640 ± 0.136	0.530 ± 0.344	0.686 ± 0.338	0.547 ± 0.149	0.422 ± 0.160
Random Forest	0.895 ± 0.023	0.847 ± 0.071	0.917 ± 0.007	0.813 ± 0.023	0.828 ± 0.040
Gradient Boosting Trees	0.889 ± 0.032	0.840 ± 0.074	0.910 ± 0.032	0.802 ± 0.073	0.818 ± 0.056

In terms of accuracy on the testing dataset, Random Forest achieved the highest accuracy (0.895 ± 0.023), followed by Gradient Boosting Trees (0.889 ± 0.032), Decision Tree (0.855 ± 0.041), Logistic Regression (0.802 ± 0.037), Gaussian NB (0.737 ± 0.033), LinearS VC (0.697 ± 0.081), Kneighbors (0.686 ± 0.020) and SGD (0.640 ± 0.136).

Based on these results, Random Forest was selected as the representative evaluation model for further analysis due to its superior performance metrics.

### The feature importance

**Figure 3:**
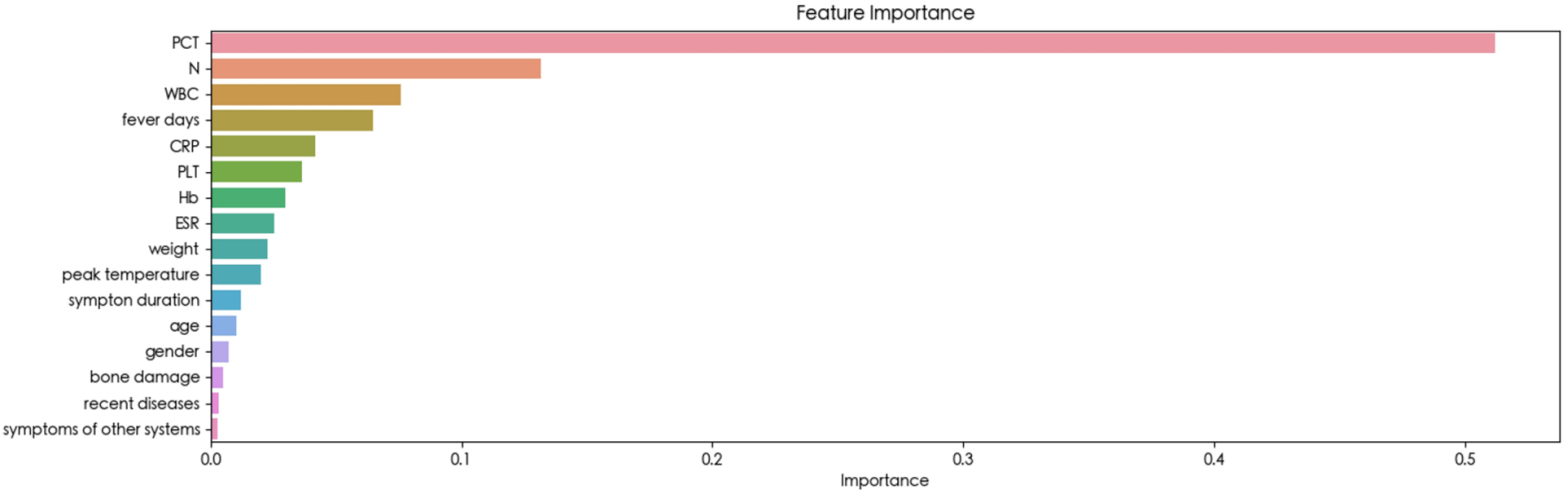
Importance matrix plot of the random forest model.

**Figure 4:**
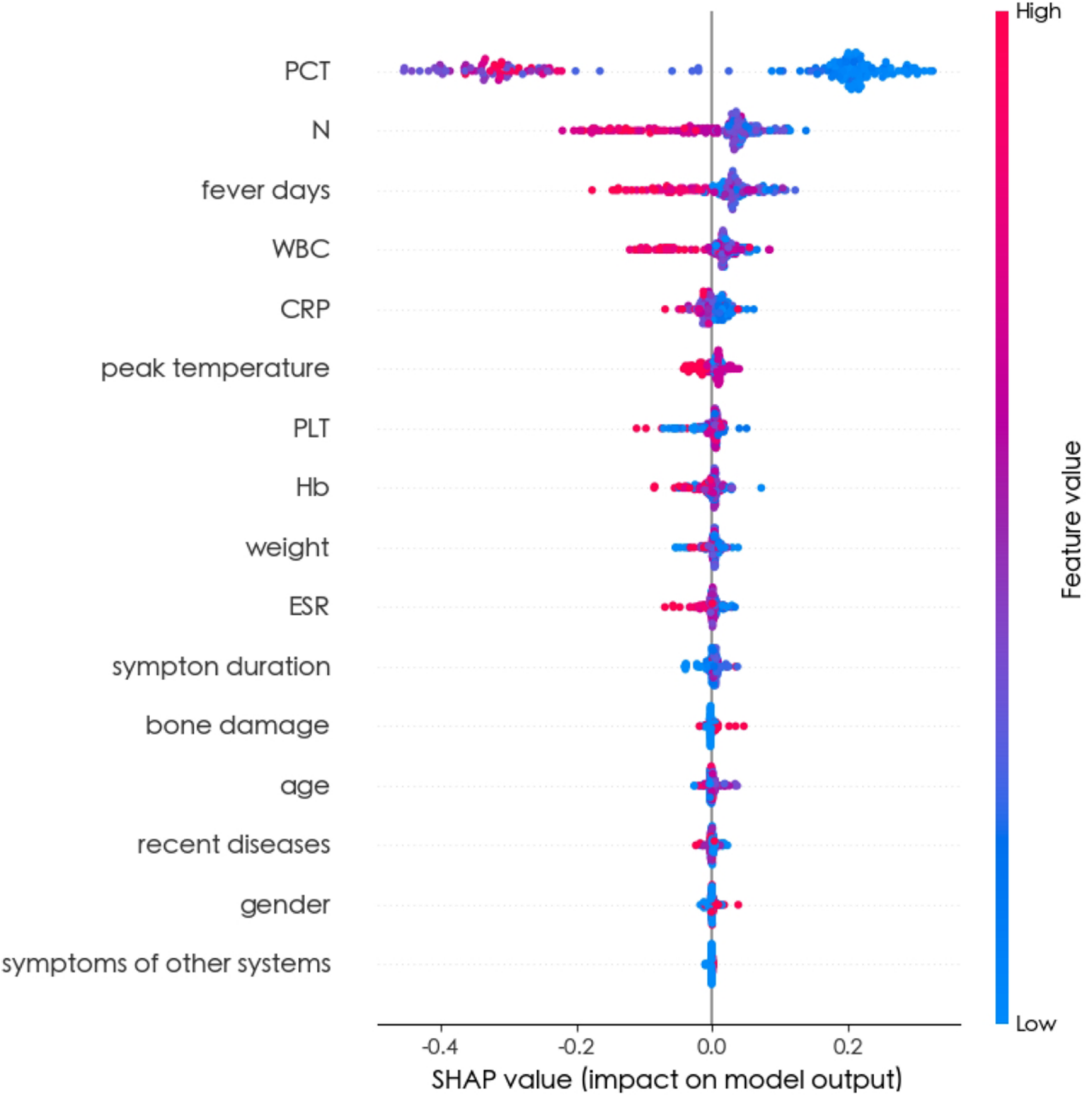
SHAP summary plot of all random forest model features. The higher the SHAP value of a feature, the higher the risk of BSI. The summary plot displayed SHAP values, which combined feature importance with feature effects. Each point on the plot represented a Shapley value for a patient's feature. The colour indicated the value of the feature, ranging from low to high. The features were ordered based on their importance.

The feature importance analysis conducted within the Random Forest model provided insights into the relevance of each variable. As depicted in Figure 3, the results revealed that PCT emerged as the most influential variable for detecting BSI. Furthermore, the SHAP summary plot of the Random Forest model and the top features predictive of BSI were illustrated in Figure 4. Notably, PCT emerged as the most important feature, with a mean SHAP value of 0.252. Both the feature importance matrix plots and SHAP summary plots identified PCT, N, WBC, and fever days as the four most crucial predictor factors for BSI.

**Figure 5:**
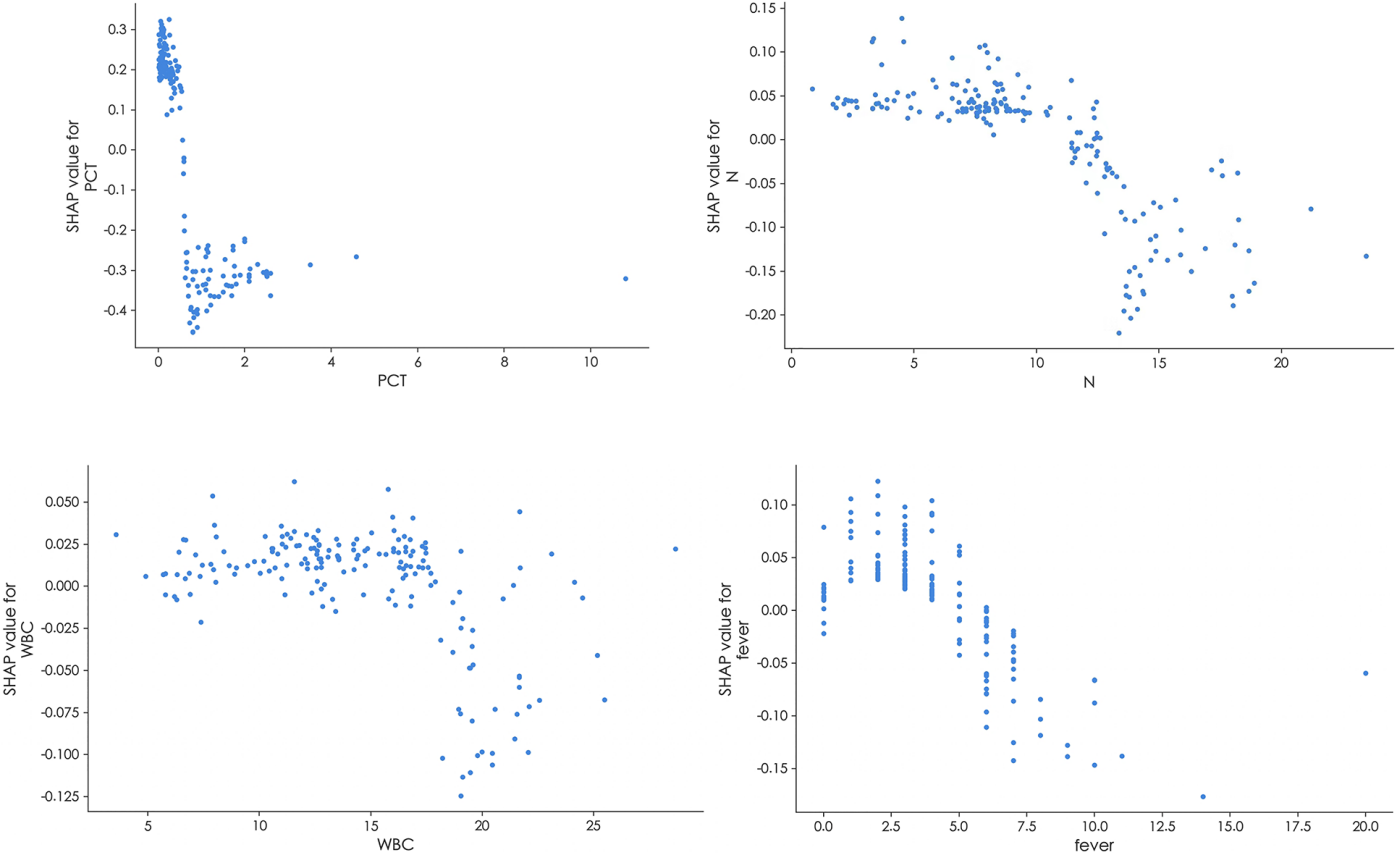
SHAP dependence plot of the random forest model. The SHAP dependence plot explains how a single feature inffuences the model's output. Feature SHAP values above 0 indicate an elevated risk of BSI.

In Figure 5, the results of the SHAP dependence plots were depicted. The *y*-axis represented the SHAP value of the feature, while the *x*-axis illustrated the changes in feature values. SHAP values above zero for specific features indicated an increased risk of BSI.

**Figure 6:**
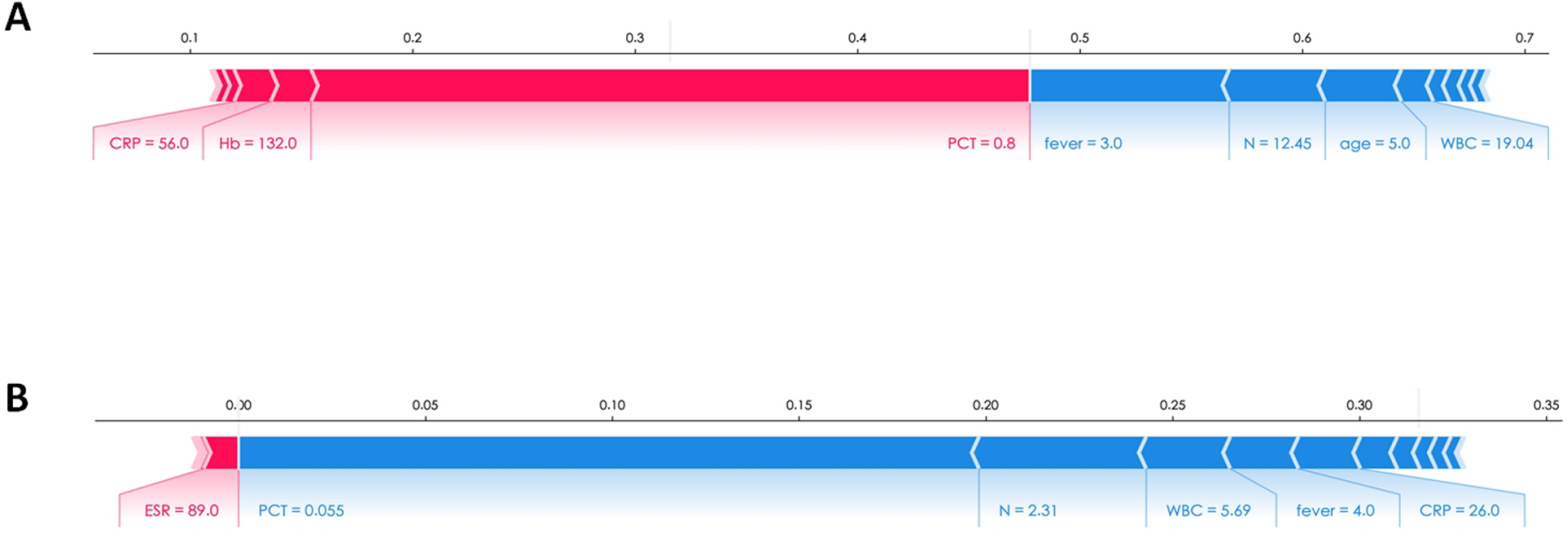
The SHAP explanation force plots of two patients. **(A)** Example of high risk according to SHAP value. **(B)** Example of low risk according to SHAP value.

The importance of these features, as provided by the SHAP value, could be visualized by the length of the bar in the SHAP dependence plots. A longer bar shape indicated a higher SHAP value, suggesting that the variable was of greater importance in influencing the model's prediction. In these plots, features that pushed the forecast higher (acting as risk factors) were displayed in red, while features that pushed the forecast lower (acting as protective factors) were displayed in blue.Two patients were selected from the test dataset, as illustrated in Figure 6. The results revealed that PCT contributed the most compared to the other variables, as indicated by the length of the bars and the colors representing the direction of influence on the forecast.

## Discussion

BSI associated with osteoarticular infections in children warrants special attention due to the vulnerability of children's immune systems and the potential for systemic infections to be overlooked. In this study, the Random Forest model demonstrated superior performance in identifying BSI associated with osteoarticular infections. This model holdes promise in aiding clinical decision-making, particularly in cases where culture results are incorrect or delayed, thereby potentially improving patient outcomes.

To the best of our knowledge, this study represented the first attempt to utilize ML algorithms for detecting BSI in children with osteoarticular infections. Sixteen clinical variables available at the time of the visit were carefully selected and utilized to train eight ML algorithms, highlighting the simplicity and convenience of facilitating timely decision-making in clinical settings. Ultimately, the Random Forest model emerged as the optimal choice, achieving the highest AUC (0.947 ± 0.016) and accuracy (0.895 ± 0.023). The feature importance matrix plot (Figure 3) and SHAP summary plot (Figure 4) further revealed that PCT, N, fever days, and WBC played significant predictive roles in identifying BSI. These findings underscore the potential of ML-based approaches in enhancing the early detection and management of BSI in pediatric patients with osteoarticular infections.

In this study, both the feature importance matrix plot and the SHAP analysis identified PCT as the most critical factor for BSI. This finding was further corroborated by the SHAP dependence plot (Figure 5), which demonstrated that as the concentration of PCT increased, the risk of BSI also increased. Previous studies have established PCT as a vital indicator of sepsis, with the majority of sepsis cases resulting from worsening BSI (20–23). Plasma PCT levels are known to be elevated in patients with severe infections such as sepsis, while they tend to be lower in patients with less severe inflammatory responses. Notably, studies have consistently demonstrated that PCT is a superior indicator for the early prediction of BSI compared to CRP (24–26), which aligns with the findings of this study.

Neutrophils are the most abundant leukocytes and play a crucial role in the immune response against infections. Dysfunctional antimicrobial effector functions and impaired migration of neutrophils have been observed in BSI (27). This may explain the high feature importance of WBC and N observed in the model training. Notably, we selected neutrophil count rather than neutrophil percentage as a variable because previous studies have demonstrated that neutrophil percentage is unreliable in predicting BSI (28). Additionally, we opted not to include the neutrophil-to-lymphocyte ratio (NLR) as a training variable due to its reliance on other biological markers to enhance diagnostic accuracy for BSI (29).

Fever is a common symptom observed in infectious diseases and can arise from both osteoarticular infections and BSI. When bacteria invade the body, the immune system initiates a cascade of defense mechanisms to combat the pathogen. These mechanisms include the inflammatory response and thermoregulation. During inflammation, various inflammatory mediators such as cytokines, chemokines, and other inflammatory molecules are released. Some of these mediators, including interleukin-1, interleukin-6, and tumor necrosis factor, can elevate the body's set point for temperature regulation by acting on the thermoregulatory center in the hypothalamus of the brain. This leads to an increase in body temperature, resulting in fever (30). Unlike localized osteoarticular infections, BSI represents a systemic state of infection where bacteria have a more widespread effect throughout the body. If left untreated, this systemic infection can persist and worsen. This broader impact of infection may explain the significance of fever days in the model training, as it reflects the duration and severity of the systemic response to infection.

Additionally, this study excluded neonatal patients, as they typically have an underdeveloped immune system and may present with atypical clinical symptoms (31). Including neonatal patients in the study could potentially introduce confounding factors that might interfere with the training of the model. Therefore, focusing solely on pediatric patients beyond the neonatal period helps ensure the consistency and reliability of the model's predictions.

For different medical conditions, specific ML algorithms may offer superior prediction accuracy. In this study, RandomForest emerged as the top-performing algorithm, demonstrating its effectiveness in feature learning. However, Random Forest typically requires a large dataset for optimal performance, as it leverages ensemble learning to improve predictive accuracy. Consequently, the inclusion of a substantial amount of data enhanced the reliability of the model's results. Future research endeavors should prioritize revalidating the model by incorporating datasets from multiple research centers. This collaborative approach can enhance the model's predictive accuracy and generalizability across diverse patient populations and clinical settings. Moreover, extending the capabilities of the model to predict specific bacterial infections would represent a valuable advancement. Such enhancements would provide early guidance for the judicious and targeted use of antibiotics, contributing to efforts aimed at combating antimicrobial resistance and improving patient outcomes.

The study has several limitations that warrant acknowledgment. Firstly, the inclusion criteria may have inadvertently excluded true-positive cases with inconsistent results due to procedural errors or other factors. Secondly, the dataset utilized in this study was limited in scope, making it difficult to make predictions about the specific bacterial species responsible for the infections. Thirdly, the applicability of the findings may be constrained in cases involving patients with multiple bacterial infections, as all positive cases included in the analysis exclusively featured a single bacterial infection.

## Conclusion

The Random Forest model constructed in this study performed well in the early detection of combined BSI in children with osteoarticular infections and may aid in clinical decision making.

## Data Availability

The original contributions presented in the study are included in the article/Supplementary Material, further inquiries can be directed to the corresponding author/s.
